# Refining the Global Picture: The Impact of Increased Resolution on CO_2_ Atmospheric Inversions Using OCO‐2 XCO_2_ Retrievals

**DOI:** 10.1029/2024JD041016

**Published:** 2024-11-23

**Authors:** Zoé Lloret, Frédéric Chevallier, Anne Cozic

**Affiliations:** ^1^ Laboratoire des Sciences du Climat et de l’Environnement LSCE/IPSL CEA‐CNRS‐UVSQ Université Paris‐Saclay Gif‐sur‐Yvette France

**Keywords:** atmospheric CO_2_, inversion, resolution, GPU

## Abstract

The threat posed by the increasing concentration of carbon dioxide (CO_2_) in the atmosphere motivates a detailed and precise estimation of CO_2_ emissions and removals over the globe. This study refines the spatial resolution of the CAMS/LSCE inversion system, achieving a global resolution of 0.7° latitude and 1.4° longitude, or three times as many grid boxes as the current operational setup. In a 2‐year inversion assimilating the midday clear‐sky retrievals of the column‐averaged dry air mole fraction of carbon dioxide (XCO_2_) from NASA's second Orbiting Carbon Observatory (OCO‐2), the elevated resolution demonstrates an improvement in the representation of atmospheric CO_2_, particularly at the synoptic timescale, as validated against independent surface measurements. Vertical profiles of the CO_2_ concentration differ slightly above 22 km between resolutions compared to AirCore profiles, and highlight differences in the vertical distribution of CO_2_ between resolutions. However, this disparity is not evident for XCO_2_, as evaluated against independent reference ground‐based observations. Global and regional estimates of natural fluxes for 2015–2016 are similar between the two resolutions, but with North America exhibiting a higher natural sink at high resolution for 2016. Overall, both inversions seem to yield reasonable estimates of global and regional natural carbon fluxes. The increase in calculation time is less than the increase in the number of operations and in the volume of input data, revealing greater efficiency of the code executed on a graphics processing unit. This allows us to make this higher resolution the new standard for the CAMS/LSCE system.

## Key Points

- We upgraded our global atmospheric inverse system to 0.7° latitude by 1.4° longitude with a modest computational overhead
- The resolution increase improves CO_2_ transport representation, benefiting coastal stations the most
- While global flux estimates in 2015–2016 were similar across resolutions, the high resolution attributes higher sinks to land than ocean

## 1 Introduction

The escalating carbon dioxide (CO_2_) concentration in the atmosphere, driven by anthropogenic emissions, is a primary catalyst for climate change. Notably, the Intergovernmental Panel on Climate Change (IPCC) estimates a global mean surface temperature increase of approximately 1.07°C during the period 2011–2019 compared to the preindustrial era (1850–1900) (IPCC et al., 2019), underscoring the urgency of addressing greenhouse gas emissions, particularly CO_2_, to damp climate variations. Precise spatiotemporal estimations of these emissions are imperative for effective mitigation strategies.

While direct measurements of carbon fluxes provide essential insights for that goal, their spatial coverage remains limited for mapping extensive regions globally. However, contemporary direct measurements of CO_2_ mole fractions are abundant in numerous regions worldwide, complemented by valuable satellite observations offering a macroscopic view of CO_2_ distribution. Leveraging this wealth of information, inverse atmospheric transport systems within a Bayesian framework enable the inference of CO_2_ sources and sinks by optimizing surface fluxes based on observed CO_2_ mole fractions and analyzed meteorological variables.

These inversions, whether conducted at a global or a regional scale, grapple with inherent uncertainties, particularly at finer scales. Notably, the Global Carbon Budget 2023 of the Global Carbon Project (Friedlingstein et al., 2023) revealed significant spread across inversions, with estimates of the net atmosphere‐to‐surface sink in the northern latitudes (>30°N) from 2013 to 2022 ranging between 1.7 and 3.3 GtC yr^−1^. Much of this spread is attributed to errors in the transport models (Basu et al., 2018). A notable limitation in the current global models employed in the Global Carbon Budget is actually their coarse horizontal resolution, averaging only 2.80° in latitude and 2.93° in longitude in the 2023 edition. The same issue was present in the v10 Model Intercomparison Project (MIP) of the second Orbiting Carbon Observatory (OCO‐2) aimed to characterize the influence of the transport model and inversion methods on flux estimates: the average resolution of all the global transport models employed in the v10 OCO‐2 MIP intercomparison was only 3.4° latitude by 4.4° longitude (Byrne et al., 2023).

Augmenting the resolution of transport models holds promise, even at a large scale (Liu et al., 2024), reducing numerical errors and thereby fostering convergence among different models (Prather et al., 2008). Increasing the horizontal resolution presents an opportunity for mitigating the representativeness error (Tolk et al., 2008). However, this effect is not universally applicable across all resolutions and does not follow a linear trend. Notably, while kilometer‐scale resolutions have demonstrated positive impacts, particularly in regions with complex terrain (Hedelius et al., 2017), the same does not hold true at the scale of hundreds of kilometers, where an increase in horizontal resolution may not necessarily diminish this error (Lin et al., 2018).

Interestingly, the few inversions driven by OCO‐2 satellite data in the Global Carbon Budget 2023 show a smaller difference between the latitudes north of 30°N and those further south in their estimates of the net atmosphere‐land flux compared to inversions driven by surface observations. This could be due to additional information obtained when using the spatially dense OCO‐2 retrievals (Friedlingstein et al., 2023), and such a benefit of the retrievals would be better exploited at higher model resolution.

The needs of the United Nations Framework Convention on Climate Change (UNFCCC), recommending the evaluation of national emission inventories compared to atmospheric inversions (IPCC et al., 2006, 2019), further reinforces the necessity of this resolution increase (Chevallier, 2021). While this makes high‐resolution targets likely in the future for most inverse systems, it remains of crucial scientific interest to judiciously evaluate the costs and benefits associated with augmenting the horizontal resolution of atmospheric models, in order to optimize computing resources, energy use, and processing times.

Indeed, resolution enhancement comes at a considerable computational cost given the intricate demands of global inverse models involving prolonged data assimilation windows, complex statistical inversion schemes, and stable atmospheric modeling under the Courant‐Friedrichs‐Lewy condition (Courant et al., 1928). This condition imposes that for a given velocity field, when the resolution of the spatial discretization increases, the time step of the simulation must be reduced to maintain stability. The quadratic growth in the size of modeled 3D atmospheric fields with horizontal resolution necessitates a judicious balance between resolution increments and expected performance gains.

The transport model used in the CO_2_ inversion system of the European operational Copernicus Atmosphere Monitoring Service (CAMS) (https://atmosphere.copernicus.eu/) underwent a first horizontal resolution increase back in 2015, doubling the number of vertical layers from 19 to 39 (Locatelli et al., 2015), and a substantial upgrade of the physic in 2018 (Remaud et al., 2018). Tests at higher spatial and vertical resolutions (another doubling of the vertical layers to 79, and a doubling of the number of horizontal boxes to reach a resolution around 2° over the whole globe) proved inadequate for accurately simulating atmospheric dynamics in regions characterized by complex topography, such as mountainous areas (Remaud et al., 2018): the increased 3D resolution did not yield a significant improvement compared to observational data, underscoring the need for further refinement, particularly to show improvement at the synoptic timescale (Agustí‐Panareda et al., 2019). The vertical profiles of CO_2_ concentration were not significantly affected by changes in resolution unlike the XCO_2_ fields, especially around emission hotspots. The high computing cost associated with this resolution increase delayed its implementation in the production chain of the CAMS CO_2_ inversion product until the code was ported on graphics processing units (GPUs) in 2023 (Chevallier et al., 2023). The migration also opened the possibility of further resolution increases while maintaining a processing time, or “time to solution,” compatible with operational constraints.

This study investigates the effect of enhancing horizontal resolution on global‐scale CO_2_ inversion to about 1°. The comparison entails evaluating the outcomes of a 2‐year inversion at an increased resolution, assimilating OCO‐2 data, against a reference configuration and independent observations. The choice of the OCO‐2 data, rather than surface or other satellite measurements, is linked to their global coverage, rapid availability, and exceptional quality, making them a backbone of low‐latency carbon cycle monitoring. The study examines both the influence of horizontal resolution on atmospheric CO_2_ transport and the overall impact on the final estimates of carbon fluxes. The subsequent section delineates the inverse system and the experimental setup, followed by a presentation of results compared to independent observations between low and high resolutions in Section 3. Section 4 succinctly summarizes the findings and concludes with insights derived from this resolution increase.

## 2 Model and Inversion Setup

### 2.1 Inversion System

The inversion system that is used to perform global CO_2_ and N_2_O atmospheric inversions for CAMS has been developed in the LSCE since 2004 (Chevallier et al., 2005). The same system has also been used outside CAMS for other tracers, such as methane (Berchet et al., 2021), carbon monoxide, or nitrogen oxides (Fortems‐Cheiney et al., 2021).

This inverse system is based on a 4D variational approach of the Bayesian inversion problem: assimilating observational data of CO_2_ concentrations to derive an optimal state of CO_2_ fluxes given a prior estimate of the CO_2_ fluxes.

Mathematically, this consists in iteratively minimizing a cost function *J*, which is defined as follows:

(1) $$J\left(x\right)=\frac{1}{2}{\left(x-x^{b}\right)}^{T}B^{-1}\left(x-x^{b}\right)+\frac{1}{2}{\left(Hx-y\right)}^{T}R^{-1}\left(Hx-y\right)$$

Here, **x** represents the vector of the variables being optimized, which, in this case, corresponds to successive global maps of the CO_2_ fluxes at the resolution of the transport model, weekly throughout the inversion window, and to the 3D state of CO_2_ at the start of the inversion window. **x** ^**b**^ means the vector of the prior state of **x**, and **y** represents the assimilated observations. The matrices **R** and **B** correspond to the error covariance matrices associated with the uncertainty of the assimilated observations, as defined from the transport model, and of the prior fluxes, respectively. The linearized operator **H** projects the control vector **x** into the observation space: it is primarily based on the transport model. In our case, the transport model is an off‐line version of the general circulation model (GCM) of the Laboratoire de Météorologie Dynamique (LMDZ) in its latest version, LMDZ6A (Hourdin et al., 2020; Remaud et al., 2018). The off‐line version only solves tracer transport equations, driven by precomputed air mass fluxes from a reference run of the full GCM nudged to the 3‐hourly horizontal winds from the fifth generation ECMWF reanalysis (ERA5). The code of the off‐line transport model corresponds to the one made public by Chevallier et al. (2023) with some memory optimizations in order to accommodate the larger arrays of the new resolution. The inversion system, coded in Python and run on CPUs, orchestrates the connection across monthly runs of the transport model, coded in Fortran and basically run on GPUs, ensuring the coherence and continuity of the inversion process.

The minimization of *J* is done iteratively by calculating its gradient using the adjoint version of the transport model and a conjugate gradient algorithm (Chevallier et al., 2005; Fisher, 1998).

### 2.2 Inversion Configuration

To assess the impact of the resolution increase on our inverse system, we conducted two global‐scale CO_2_ inversions around years 2015 and 2016, incorporating three months for spin‐up in 2014 and three months for spin‐down in 2017, at two distinct horizontal resolutions. The inversion of reference, referred to as the low‐resolution (LR) model throughout the text, operates on a latitude‐longitude grid with dimensions of 1.27° in latitude, 2.50° in longitude, and 79 vertical layers, totaling 1,626,768 cells with each cell of size 140 km by 278 km at the equator. The new resolution, designated as the high‐resolution (HR) model hereafter, utilizes a latitude‐longitude grid with dimensions of 0.70° in latitude, 1.41° in longitude, and 79 vertical layers, resulting in a total of 5,177,344 cells with each cell of size 78 km by 157 km at the equator. The model time step of the LR is 5 min for horizontal advection, 10 min for vertical advection, and 20 min for subgrid processes. In order to respect the Courant‐Friedrichs‐Lewy condition for stability in the HR, it has to go down to 3 min for horizontal advection and 6 min for vertical advection; for subgrid processes, we reduce it as well to 12 min. In both LR and HR configurations, the precomputed air mass fluxes are 3‐hourly averages.

Both inversions share identical prior states for CO_2_ fluxes, which are interpolated onto their respective grids, incorporating the following data sources with their native resolution:

● CO_2_ fluxes over the ocean are based on the CMEMS‐LSCE‐FFN 2022 estimates at a native monthly 0.25° resolution (Chau et al., 2022).
● CO_2_ biomass burning emissions are from the GFED4.1s inventory at a native monthly 0.25° resolution. No atmospheric source of CO_2_ is considered.
● CO_2_ fossil emissions are based on GCP‐GridFEDv2023.1 estimates at a native monthly 0.1° resolution (Jones et al., 2021).
● Natural fluxes of CO_2_ from the biosphere are based on a climatology of 3‐hourly averaged estimates from the ORCHIDEE model, version 2.2, and revision 7262 (Friedlingstein et al., 2022; Krinner et al., 2005) at a 0.5° resolution.

Observations of midday clear‐sky total column‐averaged CO_2_ concentrations over land from the OCO‐2 satellite were assimilated, specifically NASA's Atmospheric CO_2_ Observations from Space (ACOS) bias‐corrected nadir and glint land retrievals of XCO_2_, version 11.1 (OCO‐2/OCO‐3 Science Team et al., 2022; O’Dell et al., 2018, 2023). OCO‐2 ocean observations were not used in this study, neither were observations over mixed land‐water surfaces. Only data flagged as “good” were used, as 10‐s averages, that is, about 67 km along the orbit track, with an averaging procedure implemented at LSCE and similar to the one defined in the OCO‐2 MIP (Peiro et al., 2022). In order to account for likely correlations between the transport model errors at the subgrid scale, we deweighed the OCO‐2 binned retrievals that fall within a same LMDz grid box for a same orbit by inflating the assigned error variance by the number of retrievals in the box.

The retrievals initially adhered to the X2007 scale of the World Meteorological Organization (WMO). We converted them to the X2019 scale following Hall et al. (2021):

(2) $$X_{2019}=1.00079\cdot X_{2007}-0.142ppm$$

When assimilating the satellite retrievals, the prior and averaging kernel of each retrieval were used in the model. No other data were assimilated so that flasks and in situ and ground‐based XCO_2_ observations are fully independent.

The spatial correlations of the prior uncertainty, which drive the off‐diagonal terms of **B** in Equation 1, decay exponentially with a length of 500 km over land and 1,000 km over sea for both resolutions. The standard deviations over land are proportional to the climatological daily‐varying heterotrophic respiration flux simulated by ORCHIDEE and are constant in gC ⋅ m^−2^ per day over the ocean. They were tuned at each resolution so that over a full year, the total 1‐sigma uncertainty for the prior land fluxes amounts to 2.9 GtC ⋅ yr^−1^, and for the open ocean, to a global air‐sea flux 1‐sigma uncertainty of 0.2 GtC ⋅ yr^−1^.

Both inversions were performed over 40 iterations, on 1 CPU and 1 NVIDIA A100 GPU as in Chevallier et al. (2023). The inversion system may be accelerated with a physical parallelization in which the years are run in parallel on different GPUs with a spin‐up period for each (Chevallier, 2013), but this possibility has not been exploited here.

The inversions took 4 days and 4 hr for the LR model and 9 days and 15 hr for the HR model. This twofold increase in overall inversion computing time is much smaller than the sixfold increase in the number of operations within the transport model: threefold for the number of global grid cells and an additional twofold for the number of time steps. It is less than the extra computations induced by the ninefold increase in the dimension of the prior error covariance matrix **B**. It is also relatively less than what the threefold increase in the volume of transport model input data implies on reading time. Since the computer code is the same between the two resolutions, the relatively modest increase in calculation time reveals better efficiency of our code with increased resolution, which is not unexpected with GPUs, since higher resolutions allow larger loops that better keep the GPUs busy.

### 2.3 Evaluation

We evaluated the two inversions by directly comparing their final state and estimates of CO_2_ fluxes at the global, regional, and local scales. We also compared them to independent observations of CO_2_ concentrations.

#### 2.3.1 CO_2_ Data for Evaluation

To assess the agreement between our simulated tracer concentrations and observed data, we sampled mole fraction fields at the nearest cell center, model level (when relevant), and timestamp for each data point. We utilized high‐quality measurements from the CO_2_ GLOBALVIEWplus v8.0_2022‐08‐27 ObsPack database (ICOS RI et al., 2023; Lan et al., 2023; Miles et al., 2017; Miles et al., 2018; Schuldt et al., 2022) on the WMO CO_2_ X2019 scale (Hall et al., 2021). For AirCore, we used Version 20230831 of the data set from NOAA (Baier et al., 2021). We also exploited ground‐based XCO_2_ retrievals from the Total Carbon Column Observing Network (TCCON, Wunch et al., 2011) from which we selected in 2015 and 2016 twenty Fourier transform spectrometers around the globe (Buschmann et al., 2022, C et al., 2022, Deutscher et al., 2023; De Maziere et al., 2022; Dubey et al., 2022; Iraci et al., 2022; Kivi et al., 2022; Liu et al., 2023; Morino et al., 2022a; Morino et al., 2022b; Notholt et al., 2022; Sherlock et al., 2022; Shiomi et al., 2022; Strong et al., 2022; Sussmann & Rettinger, 2023; Te et al., 2022; Warneke et al., 2022; Wennberg et al., 2022a; Wennberg et al., 2022b; Wennberg et al., 2022c; Wunch et al., 2022).

Similar to prior studies involving inverse modeling with LMDZ, we only selected measurements that could be well modeled by a transport model, particularly avoiding tracer accumulation at low altitudes. For in situ surface stations located under 1,000 m above sea level (a.s.l.), we only considered data from 12:00 to 16:00 local time; for in situ stations above 1,000 m a.s.l., only nighttime data from 00:00 to 4:00 local time were retained. We kept all flask measurements.

The observations were categorized into three groups: surface in situ and flask measurements, AirCore flight measurements, and remote‐sensing observations from the OCO‐2 mission and TCCON sites. Vertical profiles of CO_2_ mole fraction were obtained using AirCore, an atmospheric sampling system that collects successive samples of ambient air (Baier et al., 2021; Karion et al., 2010). From the ObsPack data set, 112 surface stations were selected for analysis, excluding those with fewer than 1,200 measurement points over the 2‐year study period that passed the initial data selection criteria. The full list of ObsPack and TCCON stations used is available as a supplement. All samples from AirCore data were retained.

The uncertainty associated with the in situ and flask CO_2_ mole fraction measurements used in this study is approximately 0.1 micromol per mol (or part per million, ppm), as detailed in Crotwell et al. (2020) for systematic errors and Hazan et al. (2016) for standard deviation. This uncertainty is considered negligible compared to the model uncertainty stemming from transport errors, estimated to be around 1 ppm under 3,000 m (Lauvaux et al., 2009). The altitude determination error for AirCore measurements due to storage diffusion can be substantial, ranging from approximately 250 m below 20–1 km above that altitude (Wagenhäuser et al., 2021). The uncertainty of the measurements of the AirCore sample itself is under 0.1 ppm on average. The precision of TCCON measurements varies by site but generally remains below 0.25% (1‐sigma) for individual measurements of XCO_2_ under clear or partly cloudy skies.

**Figure 1:**
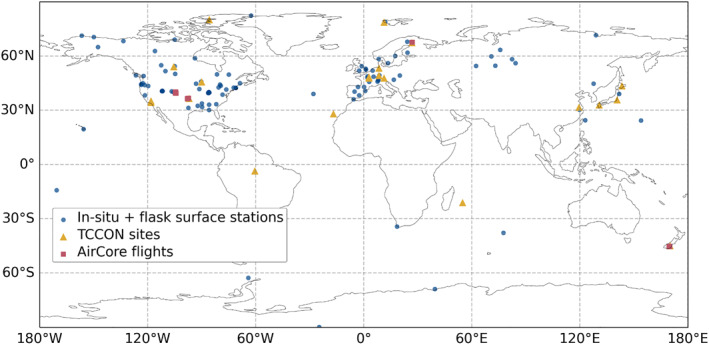
A map of the location of selected surface stations (blue dots), TCCON sites (yellow triangles), and AirCore flights (red squares).

The spatial distribution of these observation sites is as expected very unequal across the globe (Figure 1), with the majority of them situated between 30°N and 60°N. TCCON sites provide nonetheless a good overview of different latitudes, but AirCore flights for this time period are only limited to 4 different areas.

#### 2.3.2 Processing of the Surface Stations

To compare the results of our inversions with measurements from surface stations, we employed a curve‐fitting methodology to extract the annual mean, seasonal cycle, and synoptic variability of the CO_2_ mole fraction from the time series of measurements and the model. The function used for fitting consists of a second‐degree polynomial and eight harmonics (Equation 3 below). The polynomial characterizes the background growth rate in CO_2_ concentration, although this aspect is not the focus of our study due to the limited duration of our inversions. The harmonics capture the seasonal variability of CO_2_ concentrations, while the synoptic variability is obtained by subtracting the fitted curve from the raw measurements or model values (Equation 4 hereafter).

(3) $$f\left(t\right)={p_{0}+p}_{1}\cdot t+p_{2}\cdot t^{2}+\sum_{k=3}^{10}p_{k}\cdot \sin\left(2\pi kt\right)$$

(4) r(t)=x(t)−f(t)

To study the seasonal cycle, we quantify the correlation of the phase between model and measurements as well as the normalized peak‐to‐peak amplitude of the harmonics. For the synoptic variability, we look at the correlation coefficient between model and measurements and at the normalized standard deviation of the values.

The metrics are denoted by the corresponding abbreviation of the model resolution when appropriate, for example, R_LR_ for the correlation between the low‐resolution model and the measurements. When comparing a metric between the two resolutions, it is always calculated by subtracting the LR value from the HR value such as in Equation 5:

(5) $$\Delta R=R_{HR}-R_{LR}$$

The normalization of a metric in our case refers to the division of the model metric by the one of the observations. For the normalized peak‐to‐peak amplitude (NPtP) and normalized standard deviation (NSD) of the LR model, for example, in Equation 6,

(6) $${NPtP}_{LR}=\frac{{PtP}_{LR}}{{PtP}_{obs}};{NSD}_{LR}=\frac{{SD}_{LR}}{{SD}_{obs}}$$

#### 2.3.3 Processing of the Column‐Averaged CO_2_ and Vertical Profiles

In evaluating the vertical profiles of CO_2_ mole fractions, we employed a binning and averaging approach to organize the data from AirCore measurements and our models into 21 altitude bins of varied sizes between 500 m and the maximum altitude of 26 km. The height of each of these bins is shown together with the results of Figure 6 in Section 3.3. They were chosen to be more refined at the altitudes with the most differences between model and measurements.

We looked at the direct values and gradients of these vertical profiles as well as the distribution of the median bias per altitude bin.

To compare our model to independent TCCON observations on the X2019 scale, we computed the column‐averaged CO_2_ mole fraction at each observation location and time with their respective averaging kernel and prior profile. We then computed the difference between observations and models, and in particular looked at the mean bias, correlation, and normalized standard deviation (as defined in the previous subsection). In addition, we also applied the seasonal decomposition analysis described above to the TCCON observations.

#### 2.3.4 Processing of the Surface Flux Estimates

To study the regional distribution of the CO_2_ fluxes, we divided the domain into the 22 Transcom3 regions of Gurney et al. (2002) and computed the CO_2_ monthly fluxes of the two inversions in each one. From this subpartition, we also calculated annual fluxes at a global scale and a land or ocean partition.

We also compared the differences at a smaller scale by generating maps that averaged CO_2_ fluxes in each cell per season, providing insights into local variations.

## 3 Results and Discussion

### 3.1 Surface Stations

The mean correlation coefficient of the seasonal cycle across all stations studied is 0.90 for both resolutions. The average normalized peak‐to‐peak amplitude is 1.08 for the LR and 1.07 for the HR. The standard deviation for the normalized peak‐to‐peak amplitude is 0.52 for the LR and 0.42 for the HR. Both resolutions therefore capture the seasonal cycle similarly well in general, and only a few stations show large differences between the two resolutions. The HR shows a significantly lower spread of the peak‐to‐peak amplitude, indicating an improvement in modeling the seasonal variability.

**Figure 2:**
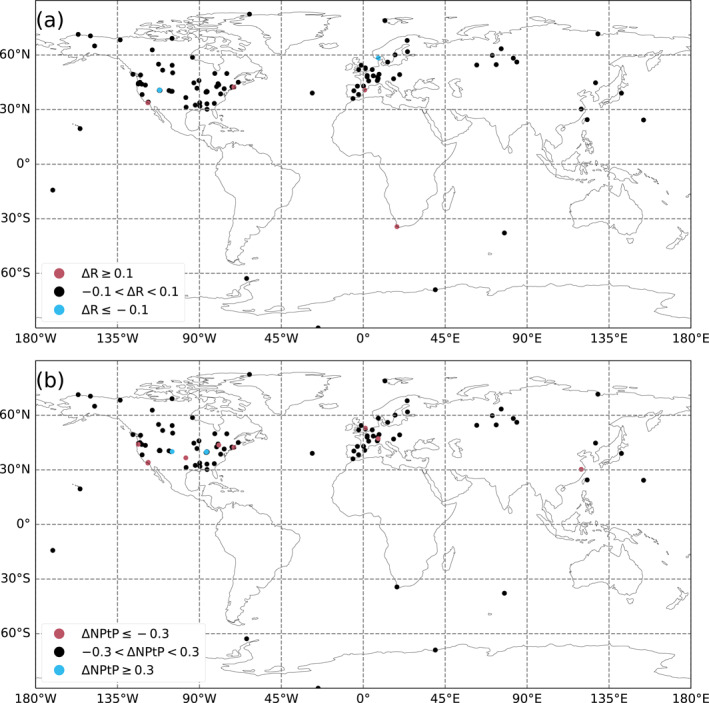
A map of the surface stations classified by Pearson correlation coefficient (a) and average normalized peak‐to‐peak amplitude (b) of the modeled versus measured CO_2_ mole fraction seasonal cycle for the years 2015–2016. Blue points correspond to stations that perform best with the LR model and red points with the HR model, according to the metrics defined in Section 2.3.2 and criteria described in Section 3.1.

The better‐performing stations in terms of seasonal cycle correlation (ΔR > 0.1) and normalized peak‐to‐peak amplitude (ΔNPtP > 0.3) for the HR model compared to the LR model are the following ones: DEC, PV, BU, CPT, SGP, CIT, BRM, OWA, WAO, LAN, and HNP. The stations that perform worse with the HR model while still capturing the seasonal cycle well in the LR model (Δ*R* < 0.1, $R_{LR}>0.7$ and ΔNPtP < 0.3, $\left|{NPtP}_{LR}-1\right|<0.5$) are as follows: BIR, UTSUG, UTMSA, BAO, INX06, and INX07. Their locations and characteristics are presented in Table 1 and shown in Figure 2.

**Table 1:** Notable Stations Identified by Seasonal and Synoptic Variability Performance

Station code	Type	Country	Seasonal better‐performing version	Synoptic better‐performing version
BAO	Urban, mountainous	USA	LR	LR
BIR	Coastal	Norway	LR	None
BRM	Mountainous	Switzerland	HR	HR
BU	Coastal, urban	USA	HR	HR
CIT	Coastal	USA	HR	HR
CPT	Coastal	South Africa	HR	None
CRV	Boreal	USA	None	LR
DEC	Coastal	Spain	HR	HR
HNP	Urban, lake	Canada	HR	HR
INU	Boreal	Canada	None	LR
INX06	Urban	USA	LR	None
INX07	Urban	USA	LR	None
LAN	Coastal, mountainous	China	HR	None
OMP	Coastal, mountainous	USA	None	HR
OWA	Coastal, mountainous	USA	HR	None
PV	Coastal	USA	HR	HR
SGP	Plains	USA	HR	HR
UTMSA	Urban	USA	LR	LR
UTSUG	Urban	USA	LR	None
WAO	Coastal, mountainous	UK	HR	HR

*Note*. A station is identified as better‐performing for a certain resolution if the difference in metric between the resolutions is superior to a threshold as defined in Section 3.1. The metrics for the seasonal cycle are the correlation and normalized peak‐to‐peak amplitude. For the synoptical variability, it is the correlation and normalized standard deviation.

There is no general trend directly linking these results to the latitude of the studied stations.

The mean synoptic variability correlation slightly improves at the higher resolution, going from 0.36 for the LR to 0.38 for the HR. The average normalized standard deviation is 1.33 for the LR model, and is reduced to 1.29 for the HR model. This shows a small but significant overall improvement regarding the synoptic variability of surface stations when increasing the resolution of our model. The improvement is actually pronounced at the lower end (mean improvement of 0.03 for $R_{LR}$ < 0.4) while correlations are hardly changing at the higher end (mean improvement of 0.002 for $R_{LR}$ > 0.4).

**Figure 3:**
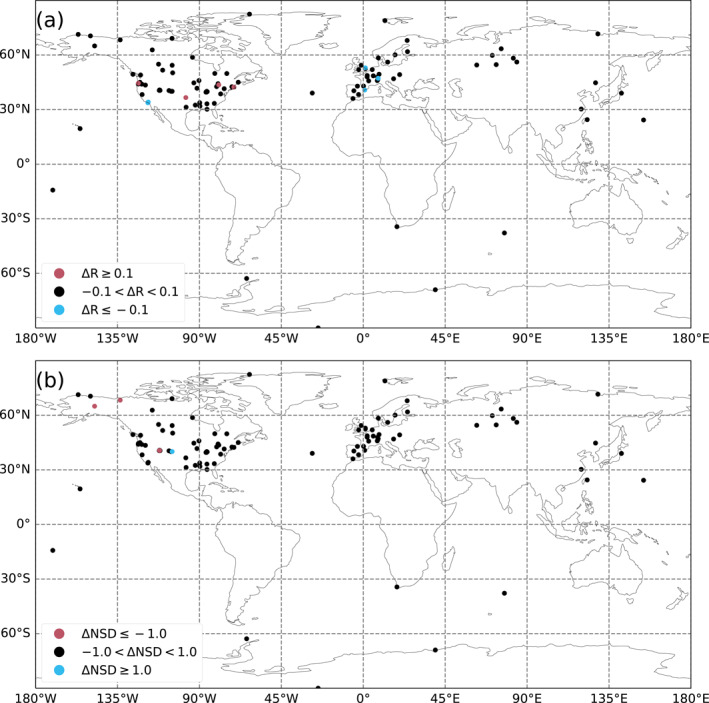
Same as Figure 2 but for the Pearson correlation coefficient (a) and the normalized standard deviation (b) of the daily average residue between our modeled and measured CO_2_ mole fraction at the surface stations averaged for the years 2015–2016.

The better‐performing stations in terms of synoptic variability correlation (Δ*R* > 0.1) and normalized standard deviation (ΔNSD > 1.0) for the HR model compared to the LR model are the following ones: DEC, PV, BU, WAO, HNP, OMP, SGP, CIT, and BRM. The stations that perform worse with the HR model while still capturing the synoptic variability well in the LR model (Δ*R* < 0.1, $R_{LR}>0.3$, ΔNSD < 1.0, and $\left|{NSD}_{LR}-1\right|<1.0$) are CRV, INU, UTMSA, and BAO. Their locations and characteristics are also presented in Table 1 and shown in Figure 3.

Most of the better‐performing stations at the HR are coastal or next to areas with sharp elevation changes, while the worse‐performing ones correspond to two cities, Salt Lake City and Indianapolis. The coastal and mountainous stations already perform better in the HR prior simulation than in the LR prior simulation (not shown), because the better coastline definition is hardly exploited in the assimilation of CO_2_ column retrievals.

### 3.2 TCCON Observations

**Figure 4:**
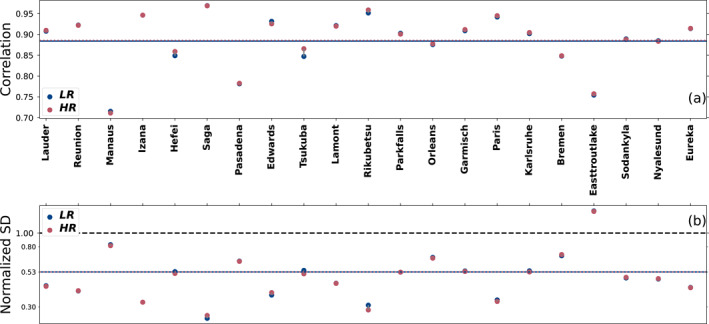
Correlation (a) and normalized standard deviation (b) of the difference between the model XCO_2_ and remotely sensed XCO_2_ from TCCON stations averaged over the years 2015–2016 for each station and then averaged across the 25 stations. Blue circles are for the LR model and red circles are for the HR model. The average correlation and normalized standard deviation for each resolution are in the corresponding color as a solid or dotted line in panels (a and b). The black dashed line in (b) corresponds to the ideal normalized standard deviation of 1. The stations are ordered on the abscissa by increasing latitudes. The *y*‐axis on panel (b) is in the log scale.

When comparing XCO_2_ between the final state of our inversion and independent observations from TCCON, we see that the mean difference between the model and observations is almost identical for both resolutions, at 0.06 ppm for the LR and 0.08 ppm for the HR (not shown). Figure 4 shows that the average correlation is 0.88 for the LR and 0.89 for the HR. The average normalized standard deviation is 0.53 for both resolutions. When looking at the behavior of individual stations, the result is very different, with both the general bias and normalized standard deviation varying widely for different stations, without any obvious link with the station location. However, both resolutions behave similarly to each other at each station, with the worst‐performing stations being identical for both resolutions. The two urban stations of Hefei and Tsukuba show a better correlation at HR, but this improvement is small to be contrasted with the relatively lower performance of some urban in situ stations at HR as shown in Section 3.1. These two TCCON stations can therefore not be taken as a general trend showing a better modeling of urban stations by the HR model.

**Figure 5:**
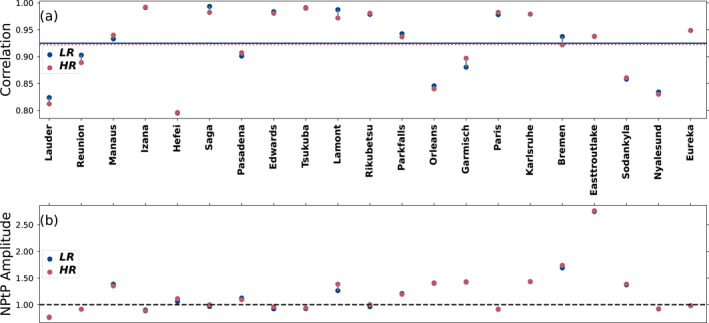
Same as Figure 4 but for the Pearson correlation coefficient (a) and the normalized peak‐to‐peak amplitude (b) of the fitted seasonal cycle between our modeled and measured XCO_2_ mole fraction at the TCCON stations averaged for the years 2015–2016.

When analyzing the seasonal fit of the observations and the model at TCCON sites in Figure 5, we see that all stations perform well in terms of correlation of the seasonal cycle, with both resolutions having a mean correlation of 0.92. They also perform almost identically regarding the modeling of the peak‐to‐peak amplitude of the seasonal cycles.

The simulation of column‐averaged CO_2_ is in principle not as sensitive to resolution increase of the transport model as for surface CO_2_ (Rayner & O’Brien, 2001), and this can explain the marginal difference between the resolutions with respect to TCCON observations.

**Figure 6:**
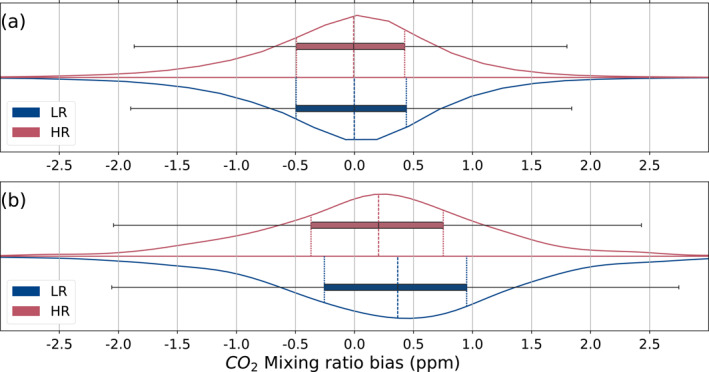
Median bias between the model and assimilated OCO‐2 observations (model minus observations) and its associated probability distribution for all available observations (a) and limited to the Transcom3 North American Boreal region (b). The curves show the kernel density estimation for each resolution (blue for LR and red for HR), and the box plots with the same colors show the median value and first and third quartiles. The whiskers correspond to one and a half of the interquartile range.

The difference in bias and standard deviation between the two resolutions compared to already assimilated OCO‐2 retrievals is negligible at the global scale with a mean bias of −0.05 ppm and standard deviation of 0.84 ppm for both resolutions shown in Figure 6a. When comparing the models to assimilated observations in only specific Transcom3 regions, we find that the only one showing significant differences between resolutions is the North American Boreal region, shown in Figure 6b. In this region, the bias of the model XCO_2_ with the assimilated OCO‐2 retrievals is significantly smaller (*t* = 5.3, *p* < 0.001) for the HR model (*M* = 0.19, SD = 0.97) compared to the LR one (*M* = 0.35, SD = 1.03).

### 3.3 Vertical Profiles

We utilized AirCore flight data to compare the CO_2_ mole fractions of our model with measurement data, obtaining vertical profiles extending to the low stratosphere. This analysis aimed to investigate the impact of increasing resolution on vertical transport. The measurements were limited in latitudes, and the results may be different in the tropics, with the majority of the measurements coming from conterminous United States (see Figure 1).

**Figure 7:**
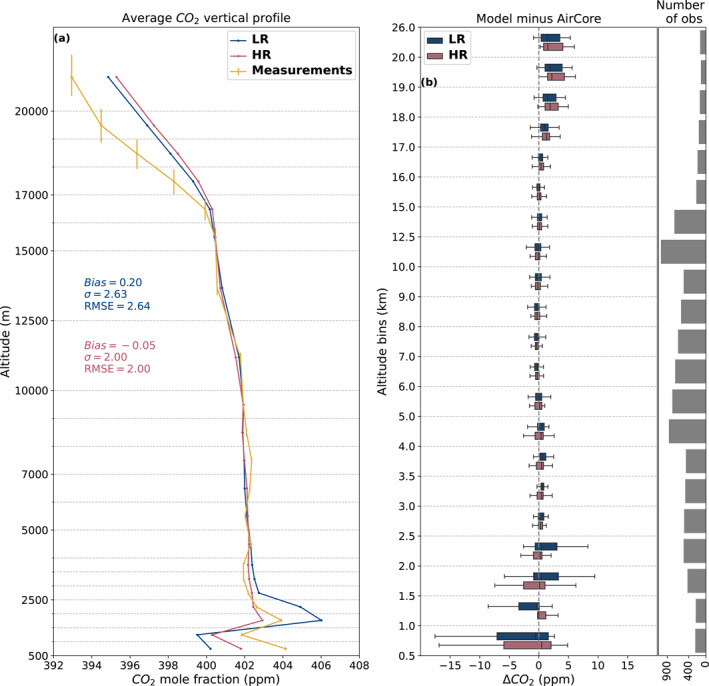
Panel (a) shows the CO_2_ mole fraction vertical profile in ppm for the two resolutions of the model (blue for LR and red for HR) and all valid AirCore sample measurements (yellow). The lines were generated by averaging the data over all altitude bins. Error bars of the measurements correspond to the altitude determination uncertainty of the sample and to the uncertainty of the measurement itself. The dotted gray horizontal lines show the altitude of each bin. The values of the bias, standard deviation, and root‐mean‐square deviation of the difference between the raw data of the models and measurements are presented for each resolution in their respective color (blue for LR and red for HR). Panel (b) shows the median bias (model minus measurements) and its probability distribution over each altitude bin averaged for all valid AirCore sample measurements. The number of measurements in each altitude bin is indicated on the right.

As depicted in Figure 7 (a), under 3 km, in and just above the boundary layer, the HR model performs better and shows a better agreement with measurements. When looking at the probability distribution of the bias at this altitude (b) we see that the HR model has a lower spread than the LR model for most altitude bins, indicating a better representation of the boundary layer. After 3 km, both resolutions of the model exhibit good agreement with measurements up to around 16 km. Beyond that, up to 22 km, both resolutions differ from measurements, showing a positive bias. This leads to a lower general bias for the HR model compared to measurements (−0.05 vs. 0.20 ppm) and a lower spread of the difference between the model and measurements (standard deviation of 2.00 vs. 2.63 ppm).

**Figure 8:**
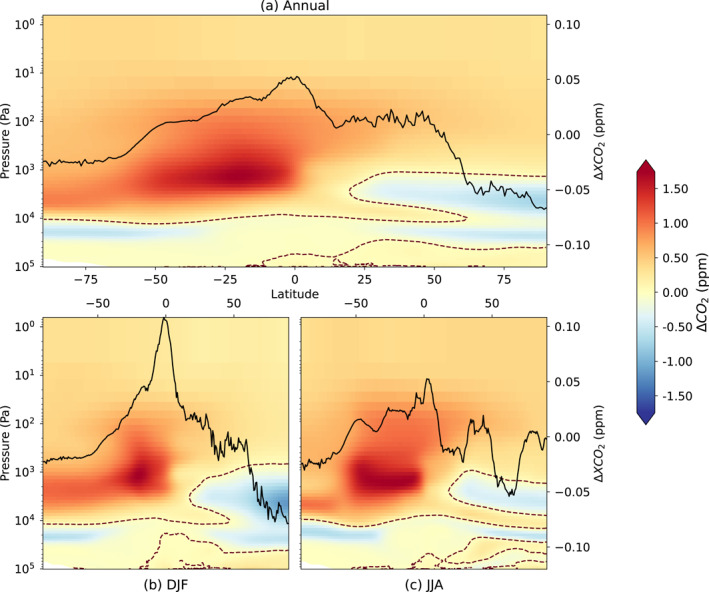
Difference in CO_2_ mole fraction in ppm between the HR and LR models after inversion (HR‐LR), averaged per month over the 2 years and per longitude band. The results are then averaged again either annually (a) or per season: DJF (b) and JJA (c). The data of the LR model were interpolated on the latitudes of the HR model before computing the difference. The dark brown dashed contour line shows places where the value of the difference equals zero. In each panel, the black line corresponds to the difference in XCO_2_ mole fraction in ppm between the HR and LR models averaged and interpolated in time and space in the same manner. The scale of this difference (HR‐LR) in ppm is on the right *y*‐axis.

When looking at the time‐averaged zonal vertical profiles of CO_2_ mole fraction, we can see that the distribution is different between the resolutions and is on the order of −0.7 to +1.7 ppm (Figure 8). These variations vary both in latitude and in altitude, and the previous comparison to AirCore data only gave a limited view into these differences. The HR model shows a higher concentration of CO_2_ in the upper atmosphere in general, but at these high altitudes, the total mass of CO_2_ is very low. The black lines in Figure 8 corresponding to the zonal mean of the difference in XCO_2_ between the resolutions show that this vertically integrated difference behaves very differently depending on the latitude and season. The difference in XCO_2_ is most important in the −25°–0° latitude band, particularly in winter. For other latitudes, the concentration difference in the high atmosphere is largely compensated by an opposite difference at lower altitudes. This suggests that in these cases, the difference in vertical profile of atmospheric CO_2_ is mostly driven by a higher vertical transport speed in our HR model, whereas around the 0° latitude band, the higher atmospheric CO_2_ concentration is present irrespective of altitude.

**Figure 9:**
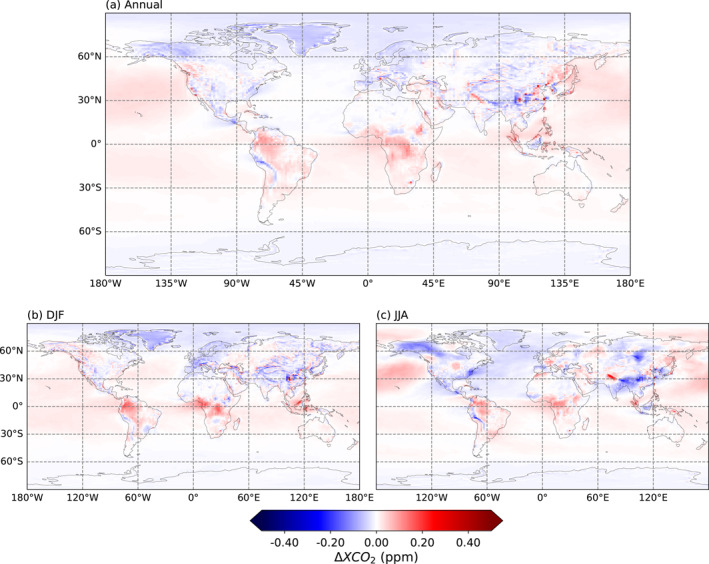
Maps of the difference in XCO_2_ mole fraction in ppm between the HR and LR models (HR‐LR) after the inversion, averaged per year and season over the 2015–2016 period. The data of the LR model were interpolated on the longitudes and latitudes of the HR model before computing the difference.

Figure 9 shows the 2D spatial distribution of the difference in XCO_2_ between the two resolutions. The difference remains small and mostly under 0.2 ppm, but a pattern still emerges. All year round, the HR model has a higher XCO_2_ mole fraction over both land and ocean in the tropics than the LR model but a lower one over land in the high northern latitudes. The overall sign opposition between northern extratropical lands and the extratropical Pacific ocean may be favored by the exclusion of ocean satellite retrievals from the assimilation system (see Section 2.2).

### 3.4 Regional Fluxes

Table 2 shows the global estimates of natural carbon fluxes after inversion for our model at both resolutions. The results are very similar to each other for both years at the global scale, with the HR model giving a slightly stronger sink. This is due to a stronger land sink at the HR, which is not fully compensated by the relatively weaker ocean sink. This difference in the total land flux is not equally distributed across space.

**Table 2:** Estimation of the Natural Carbon Fluxes per Year After Inversion for Each Model Resolution, at the Global Scale and Partitioned by Land and Ocean

Year	Model	Land flux (GtC yr^−1^)	Ocean flux (GtC yr^−1^)	Global flux (GtC yr^−1^)
2015	LR	−1.29	−1.94	−3.22
	HR	−1.54	−1.77	−3.31
2016	LR	−1.53	−2.18	−3.71
	HR	−1.87	−2.00	−3.88

Global flux estimates are in line with estimates from atmospheric inversion results using the v9 OCO‐2 retrievals for 2015 (Peiro et al., 2022), but this is true at the global scale because the land sink is higher while the ocean sink is lower. This trend is similar in 2016, but the lower ocean sink makes the global sink lower than the range of v9 OCO‐2 estimates (Peiro et al., 2022).

**Figure 10:**
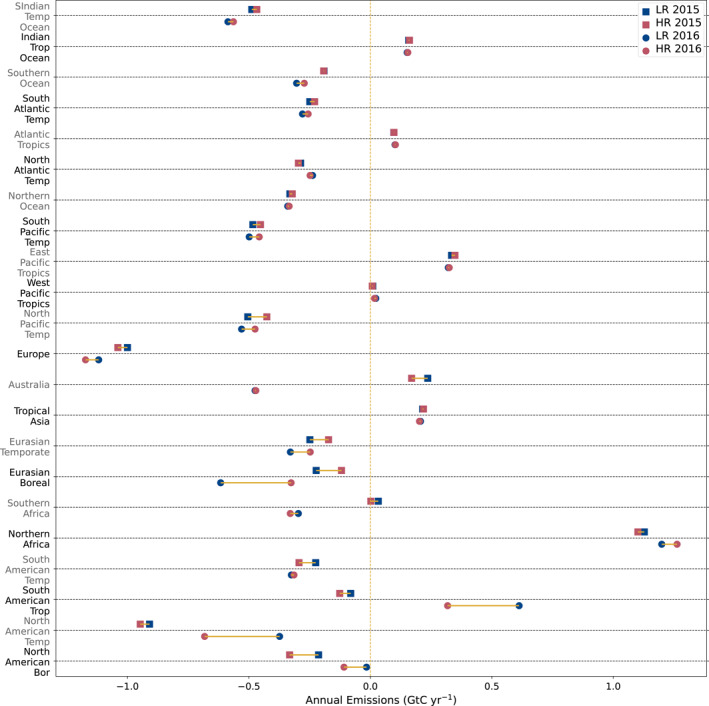
Total annual surface emissions minus the fossil fuel emissions for LR and HR (in blue and red, respectively) in GtC per year for each Transcom3 region, for the year 2015 with squares above the black dotted line, and with circles for the year 2016 below the line.

**Figure 11:**
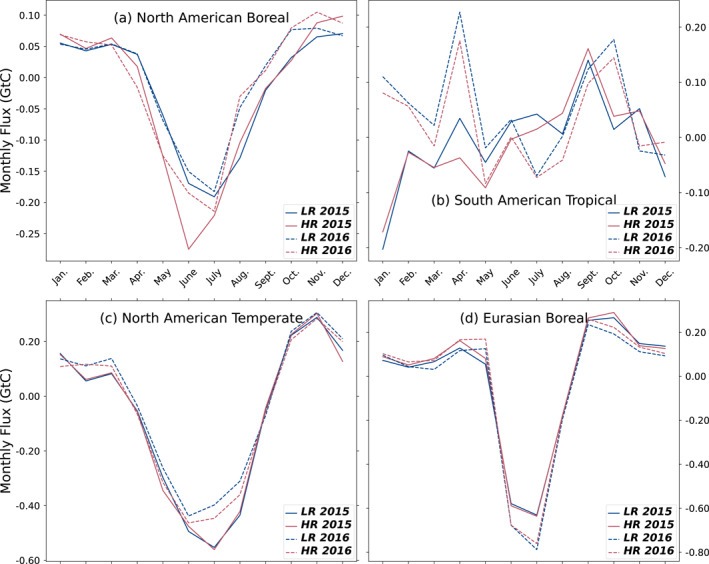
Monthly averaged surface flux minus the fossil fuel emissions for LR and HR models in GtC per month (blue and red, respectively), for 2015 and 2016 (solid lines and dashed lines, respectively) in Transcom3 regions North American Boreal (a), South American Tropical (b), North American Temperate (c), and Eurasian Boreal (d). These regions show the greatest relative difference in estimated annual flux between the two resolutions of our model.

Figure 10 shows the annual net surface flux in GtC per year minus the fossil fuel emissions per Transcom3 region for each year of our inversion and both resolutions. This information, combined with some monthly estimates of CO_2_ fluxes from Figure 11 inform us about when and where surface fluxes estimated by the inversions differ depending on the corresponding model resolution.

A few Transcom3 regions exhibit notable differences in CO_2_ flux dynamics (Figure 11). This is particularly the case for North American Boreal forests, where the HR model suggests substantially more sink in both years. This difference in regional carbon flux between the two models is not paralleled by notable discrepancies in the seasonal cycle of CO_2_ concentrations compared to independent measurements from surface stations. The CRV and INU stations situated in this region only perform worse with the HR model in terms of synoptical variability, not seasonality (as noted in Section 3.1). The results shown back in Figure 6b indicate a more efficient assimilation of the satellite data in this region. For the South American Tropical and North American Temperate regions, the HR model has a bigger carbon sink, particularly in the year 2016. This is in line with the higher global land sink of the HR model. The Eurasian Boreal region on the opposite has higher emissions in the HR model. Figure 11d shows that this is only limited to the beginning and end of a year. And given the large size of this region, the overall impact at the global scale is minor.

### 3.5 Local Fluxes

**Figure 12:**
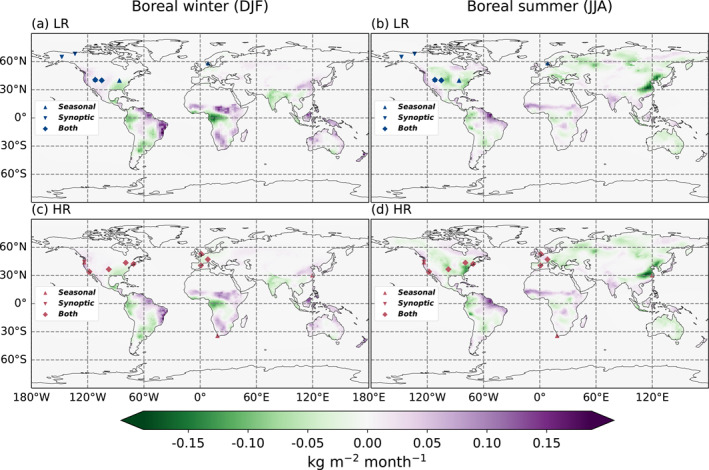
Surface flux increments between the prior and posterior state of the inversion for the LR (a and b) and HR (c and d) versions, in kg/m^2^/month. The fluxes are averaged over the corresponding months for the 2 years of inversion. December, January, and February (a and c), June, July, and August (b and c). The dots correspond to the surface stations that each resolution improve the most compared to the other one in terms of the seasonal cycle and synoptic variability, as listed in Table 1 (blue for stations performing better in LR and red for HR).

When looking at fluxes at the local scale, we can directly see the benefit of the high resolution with respect to coastal definition, in particular, in areas with complex coastlines. Figure 12 shows maps of the increments of the surface fluxes, that is, the correction of the prior fluxes by the posterior ones, averaged for winter and summer between 2015 and 2016. Some regional scale patterns discussed in Section 3.5 can be immediately seen, such as the higher summer sink of carbon for the HR model in boreal North America. The general patterns of surface fluxes for the HR model are similar to the LR model but provide much more spatial details.

The surface stations in which the HR model fits better and therefore that benefit the most from the increased resolution as discussed in Section 3.1 are situated either in continental North America, near large population centers with complex orography, or near the coast (listed in Table 1 and visible in Figure 12). This indicates that the improvement we see is not primarily caused by fine‐scale changes in the seasonal flux patterns but more so by the improved orography and wind fields used to drive the model.

**Figure 13:**
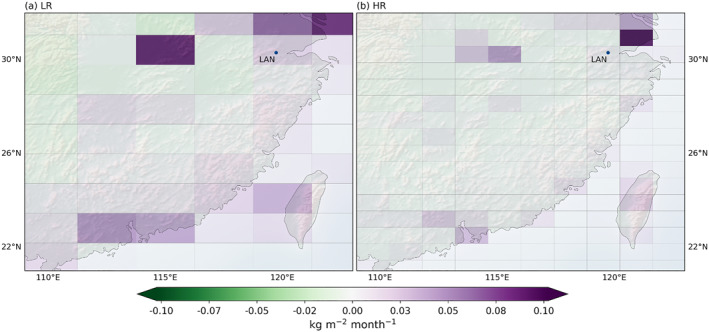
Total monthly surface flux including fossil fuel emissions averaged over the period 2015–2016 for the LR (a) and HR (b) versions, in kg/m^2^/month, zoomed around the area near the station LAN in China. The lines show the edge of the cells of each model, highlighting the difference in resolution, particularly along the coastline.

The zoom of Figure 13 exemplifies the improvement gained by the increase in resolution around Southeast China and Taiwan. The Taiwan Strait at HR is represented with some pure marine pixels in contrast to LR. Conversely, the LAN station in the northeast of the figure is in a mixed cell at LR with both land and sea surfaces, but is clearly inland at HR. Such a behavior can be seen across the globe in particular around large islands or straits. This benefit from the HR model does not come through a better assimilation of the OCO‐2 data, but is inherent to the resolution of the transport model itself.

## 4 Conclusion

We successfully increased the resolution of the CAMS/LSCE inversion system, tripling the number of global grid points and reaching a global resolution of 0.7° latitude and 1.4° longitude. This was made possible, thanks to recent developments in the model, allowing it to run on GPUs and limiting the necessary higher computational cost than the previous resolution to twice without increasing the number of devices. While this study focused on an inversion over 2 years and only assimilating OCO‐2 data over land, larger and longer‐lasting inversions are now possible and will be part of future operational work within CAMS.

As seen in the previous sections, the increase in resolution of our inverse model leads to a small but significant overall improvement in the representation of atmospheric CO_2_ compared to independent measurements from surface stations, particularly at the synoptic timescale. The stations where the benefit of the new resolution is seen the most were situated primarily near coasts or large cities. This gain was primarily due to the resolution increase of the transport model, leading to a better orography and coastal definition. This is promising for the quality of future surface‐driven inversions run at the new resolution.

The vertical profiles of CO_2_ concentration are different between the two resolutions when compared to AirCore measurements, particularly for altitudes above 22 km. The HR model performs better under 3 km, which leads to a lower general bias and spread of the difference with measurements. This difference can also be seen when looking at zonal averages of the vertical profile of CO_2_. This disparity between resolutions is, however, not evidenced when looking at XCO_2_ globally, whether when comparing the final inversion product to already assimilated OCO‐2 observations or to independent TCCON observations.

The global and regional estimates of the natural fluxes for the years 2015 and 2016 are similar for our two resolutions, but the HR model shows a consistently higher land sink and lower ocean sink than the LR model (without assimilating satellite ocean retrievals). The largest regional difference is a higher natural sink in North America for the HR model during the year 2016. Both inversions offer valid options for global and regional estimates of natural carbon fluxes, and we cannot directly demonstrate the expected superiority of the higher resolution ones.

Further enhancement in horizontal resolution holds the potential for increased benefits in atmospheric transport, with a critical threshold being the attainment of full cloud resolution rather than relying on subgrid parameterization (Schneider et al., 2017). Upcoming missions such as the Carbon Dioxide Monitoring (CO2M) and the Global Observing SATellite for Greenhouse gases and Water cycle (GOSAT‐GW) will use wide‐swath sensors, which will provide a much higher observation density. How well higher resolution inverse models will be able to leverage this increase in observation density is still not clear, the demonstrated better assimilation of OCO‐2 data by our HR model being only restricted to a limited area in the North American Boreal region. Furthermore, conventional latitude‐longitude grids may encounter computing bottlenecks when scaling up in resolution, particularly due to clustering issues at the poles. The proposed strategy for the CAMS/LSCE inversion system to address this challenge involves adopting a new dynamical core operating on an icosahedral grid (Dubos et al., 2015). Ongoing development efforts aim to bring such a core in the CAMS/LSCE inversion system in order to reach subdegree resolutions.

## Conflict of Interest

The authors declare no conflicts of interest relevant to this study.

## Supporting information

Figure S1

## Data Availability

The LMDZ off‐line transport model v3.1 is publicly available from https://doi.org/10.5281/zenodo.7324039 (Chevallier, 2022) under the Creative Commons Attribution 4.0 International License. The inverse system in Python is available as part of the CIF at https://git.nilu.no/VERIFY/CIF.

## Appendix A Observation Datasets

Table A1 presents the data sets used from the ObsPack database as well as the corresponding abbreviated site code for each station used in the main text.

**Table A1:** List of Data Sets Used From ObsPack for Surface Stations

Site code	Data set
AirCoreNOAA	aircorenoaa_aircore_1_allvalid
ABT	abt_surface‐insitu_6_allvalid
ALT	alt_surface‐flask_426_representative
ALT	alt_surface‐insitu_6_allvalid
ALT	alt_surface‐flask_1_representative
ALT	alt_surface‐flask_2_representative
ALT	alt_surface‐flask_4_representative
AMS	ams_surface‐flask_1_representative
AMS	ams_surface‐insitu_11_allvalid
AMT	amt_tower‐insitu_1_allvalid‐30magl
AMT	amt_tower‐insitu_1_allvalid‐12magl
AMT	amt_surface‐pfp_1_allvalid‐107magl
AMT	amt_tower‐insitu_1_allvalid‐107magl
AZV	azv_tower‐insitu_20_allvalid‐29magl
AZV	azv_tower‐insitu_20_allvalid‐50magl
BAO	bao_tower‐insitu_1_allvalid‐100magl
BAO	bao_tower‐insitu_1_allvalid‐300magl
BAO	bao_surface‐pfp_1_allvalid‐300magl
BAO	bao_tower‐insitu_1_allvalid‐22magl
BCK	bck_surface‐insitu_6_allvalid
BIR	bir_surface‐insitu_56_allvalid
BRA	bra_surface‐insitu_6_allvalid
BRM	brm_tower‐insitu_49_allvalid‐12magl
BRM	brm_tower‐insitu_49_allvalid‐72magl
BRM	brm_tower‐insitu_49_allvalid‐45magl
BRM	brm_tower‐insitu_49_allvalid‐212magl
BRM	brm_tower‐insitu_49_allvalid‐132magl
BRW	brw_surface‐insitu_1_allvalid
BRW	brw_surface‐flask_4_representative
BRW	brw_surface‐flask_1_representative
BRW	brw_surface‐flask_426_representative
BRZ	brz_tower‐insitu_20_allvalid‐20magl
BRZ	brz_tower‐insitu_20_allvalid‐80magl
BRZ	brz_tower‐insitu_20_allvalid‐5magl
BRZ	brz_tower‐insitu_20_allvalid‐40magl
BSD	bsd_tower‐insitu_160_allvalid‐108magl
BSD	bsd_tower‐insitu_160_allvalid‐248magl
BSD	bsd_tower‐insitu_160_allvalid‐42magl
BU	bu_surface‐insitu_59_allhours
CBW	cbw_tower‐insitu_445_allvalid‐27magl
CBW	cbw_tower‐insitu_445_allvalid‐67magl
CBW	cbw_tower‐insitu_445_allvalid‐127magl
CBW	cbw_tower‐insitu_445_allvalid‐207magl
CBY	cby_surface‐insitu_6_allvalid
CHL	chl_surface‐insitu_6_allvalid
CIT	cit_surface‐insitu_115_allhours‐200magl
COP	cop_tower‐insitu_59_allhours
CPS	cps_surface‐insitu_6_allvalid
CPT	cpt_surface‐flask_1_representative
CPT	cpt_surface‐insitu_36_marine
CRV	crv_tower‐insitu_1_allvalid‐32magl
CRV	crv_surface‐pfp_1_allvalid‐32magl
CRV	crv_tower‐insitu_1_allvalid‐17magl
CRV	crv_tower‐insitu_1_allvalid‐5magl
DEC	dec_surface‐insitu_431_allvalid
DEM	dem_tower‐insitu_20_allvalid‐45magl
DEM	dem_tower‐insitu_20_allvalid‐63magl
EEC	eec_surface‐insitu_431_allvalid
EGB	egb_surface‐insitu_6_allvalid
ENA	ena_surface‐insitu_64_allvalid‐10magl
ESP	esp_surface‐flask_2_representative
ESP	esp_surface‐insitu_6_allvalid
EST	est_surface‐insitu_6_allvalid
ETL	etl_surface‐insitu_6_allvalid
FSD	fsd_surface‐insitu_6_allvalid
GCI01	gci01_tower‐insitu_60_allvalid
GCI02	gci02_tower‐insitu_60_allvalid
GCI03	gci03_tower‐insitu_60_allvalid
GCI04	gci04_tower‐insitu_60_allvalid
GCI05	gci05_tower‐insitu_60_allvalid
GIC	gic_surface‐insitu_431_allvalid
GIF	gif_surface‐insitu_11_allvalid
GOULD	gould_shipboard‐insitu_1_allvalid
HDP	hdp_surface‐insitu_3_nonlocal
HEI	hei_surface‐insitu_22_allvalid
HFM	hfm_tower‐insitu_59_allhours
HNP	hnp_surface‐insitu_6_allvalid
HTM	htm_tower‐insitu_424_allvalid‐70magl
HTM	htm_tower‐insitu_424_allvalid‐30magl
HTM	htm_tower‐insitu_424_allvalid‐150magl
HUN	hun_tower‐insitu_35_allvalid‐48magl
HUN	hun_tower‐insitu_35_allvalid‐10magl
HUN	hun_tower‐insitu_35_allvalid‐115magl
HUN	hun_tower‐insitu_35_allvalid‐82magl
HUN	hun_surface‐flask_1_representative
INU	inu_surface‐insitu_6_allvalid
INX01	inx01_surface‐insitu_60_allhours
INX02	inx02_surface‐insitu_60_allhours
INX03	inx03_surface‐insitu_60_allhours
INX04	inx04_surface‐insitu_60_allhours
INX06	inx06_surface‐insitu_60_allhours
INX07	inx07_surface‐insitu_60_allhours
INX08	inx08_surface‐insitu_60_allhours
INX09	inx09_surface‐insitu_60_allhours
INX10	inx10_surface‐insitu_60_allhours
INX11	inx11_surface‐insitu_60_allhours
INX13	inx13_surface‐insitu_60_allhours
JFJ	jfj_surface‐insitu_5_allvalid
JFJ	jfj_surface‐insitu_49_allvalid
KAS	kas_surface‐insitu_53_allvalid
KCMP	kcmp_tower‐insitu_102_allhours‐200magl
KRS	krs_tower‐insitu_20_allvalid‐67magl
KRS	krs_tower‐insitu_20_allvalid‐35magl
LAN	lan_surface‐insitu_33_allvalid
LEF	lef_tower‐insitu_1_allvalid‐244magl
LEF	lef_tower‐insitu_1_allvalid‐122magl
LEF	lef_surface‐pfp_1_allvalid‐396magl
LEF	lef_tower‐insitu_1_allvalid‐30magl
LEF	lef_tower‐insitu_1_allvalid‐11magl
LEF	lef_tower‐insitu_1_allvalid‐76magl
LEF	lef_tower‐insitu_1_allvalid‐396magl
LEF	lef_surface‐pfp_1_allvalid‐244magl
LFS	lfs_surface‐insitu_33_allvalid
LLB	llb_surface‐insitu_6_allvalid
LLB	llb_surface‐flask_1_representative
MBO	mbo_surface‐pfp_1_allvalid‐11magl
MBO	mbo_surface‐insitu_1_allvalid‐11magl
MLO	mlo_surface‐flask_1_representative
MLO	mlo_surface‐flask_4_representative
MLO	mlo_surface‐flask_426_representative
MLO	mlo_surface‐flask_2_representative
MLO	mlo_surface‐insitu_1_allvalid
MNM	mnm_surface‐insitu_19_representative
MRC	mrc_surface‐pfp_1_allvalid‐south
MRC	mrc_tower‐insitu_60_allvalid‐south
MRC	mrc_surface‐pfp_1_allvalid‐east
NOR	nor_tower‐insitu_424_allvalid‐59magl
NOR	nor_tower‐insitu_424_allvalid‐100magl
NOR	nor_tower‐insitu_424_allvalid‐32magl
NOY	noy_tower‐insitu_20_allvalid‐43magl
NOY	noy_tower‐insitu_20_allvalid‐21magl
NWR	nwr_surface‐pfp_1_allvalid‐3magl
NWR	nwr_surface‐insitu_3_nonlocal
NWR	nwr_surface‐flask_1_representative
OLI	oli_surface‐insitu_64_allvalid‐10magl
OMP	omp_surface‐insitu_68_allhours
ONG	ong_surface‐insitu_68_allhours
OPE	ope_tower‐insitu_11_allvalid‐120magl
OSI	osi_tower‐insitu_68_allhours‐269magl
OSI	osi_tower‐insitu_68_allhours‐31magl
OWA	owa_surface‐insitu_68_allhours
PAL	pal_surface‐flask_1_representative
PAL	pal_surface‐insitu_30_nonlocal
PAL	pal_surface‐insitu_30_continental
PAL	pal_surface‐insitu_30_marine
PDM	pdm_surface‐flask_11_representative
PDM	pdm_surface‐insitu_11_allvalid
PRS	prs_surface‐insitu_21_allvalid
PUY	puy_surface‐insitu_11_allvalid
PV	pv_surface‐insitu_115_allhours‐200magl
RGL	rgl_tower‐insitu_160_allvalid‐45magl
RGL	rgl_tower‐insitu_160_allvalid‐90magl
RYO	ryo_surface‐insitu_19_representative
SCT	sct_tower‐insitu_1_allvalid‐61magl
SCT	sct_surface‐pfp_1_allvalid‐305magl
SCT	sct_tower‐insitu_1_allvalid‐305magl
SCT	sct_tower‐insitu_1_allvalid‐31magl
SGP	sgp_surface‐insitu_64_allvalid‐60magl
SGP	sgp_surface‐flask_1_representative
SMO	smo_surface‐flask_426_representative
SMO	smo_surface‐flask_1_representative
SMO	smo_surface‐insitu_1_allvalid
SMO	smo_surface‐flask_4_representative
SMR	smr_tower‐insitu_421_allvalid‐67magl
SMR	smr_tower‐insitu_421_allvalid‐17magl
SMR	smr_tower‐insitu_421_allvalid‐125magl
SNP	snp_surface‐insitu_1_allvalid‐10magl
SNP	snp_surface‐insitu_1_allvalid‐5magl
SNP	snp_surface‐insitu_1_allvalid‐17magl
SPL	spl_surface‐insitu_3_nonlocal
SPO	spo_surface‐flask_4_representative
SPO	spo_surface‐flask_2_representative
SPO	spo_surface‐insitu_1_allvalid
SPO	spo_surface‐flask_426_representative
SPO	spo_surface‐flask_1_representative
SSC	ssc_surface‐insitu_431_allvalid
SSL	ssl_surface‐insitu_107_allvalid
SYO	syo_surface‐insitu_8_allvalid
SYO	syo_surface‐flask_1_representative
TAC	tac_tower‐insitu_160_allvalid‐185magl
TAC	tac_surface‐flask_1_representative
TAC	tac_tower‐insitu_160_allvalid‐54magl
TAC	tac_tower‐insitu_160_allvalid‐100magl
TIK	tik_surface‐insitu_30_allvalid
TIK	tik_surface‐flask_1_representative
TPD	tpd_surface‐insitu_6_allvalid
TRN	trn_tower‐insitu_11_allvalid‐180magl
UTDBK	utdbk_tower‐insitu_432_allvalid
UTMSA	utmsa_tower‐insitu_432_allvalid
UTRPK	utrpk_tower‐insitu_432_allvalid
UTSUG	utsug_tower‐insitu_432_allvalid
UTUOU	utuou_tower‐insitu_432_allvalid
VAC	vac_surface‐insitu_431_allvalid
VGN	vgn_tower‐insitu_20_allvalid‐42magl
VGN	vgn_tower‐insitu_20_allvalid‐85magl
WAO	wao_surface‐insitu_13_allvalid
WBI	wbi_tower‐insitu_1_allvalid‐31magl
WBI	wbi_tower‐insitu_1_allvalid‐99magl
WBI	wbi_tower‐insitu_1_allvalid‐379magl
WBI	wbi_surface‐pfp_1_allvalid‐379magl
WGC	wgc_tower‐insitu_1_allvalid‐483magl
WGC	wgc_surface‐pfp_1_allvalid‐91magl
WGC	wgc_tower‐insitu_1_allvalid‐91magl
WGC	wgc_tower‐insitu_1_allvalid‐30magl
WGC	wgc_surface‐pfp_1_allvalid‐483magl
WKT	wkt_tower‐insitu_1_allvalid‐244magl
WKT	wkt_tower‐insitu_1_allvalid‐62magl
WKT	wkt_tower‐insitu_1_allvalid‐457magl
WKT	wkt_tower‐insitu_1_allvalid‐30magl
WKT	wkt_tower‐insitu_1_allvalid‐122magl
WKT	wkt_surface‐pfp_1_allvalid‐122magl
WKT	wkt_tower‐insitu_1_allvalid‐9magl
WKT	wkt_surface‐pfp_1_allvalid‐457magl
YON	yon_surface‐insitu_19_representative
ZEP	zep_surface‐insitu_56_allvalid
ZEP	zep_surface‐flask_1_representative

Table A2 presents in a similar way the list of TCCON sites used in the study.

**Table A2:** List of TCCON Sites Used and Their Locations

TCCON code	Location
br	Bremen, Germany
ci	Pasadena, California, USA
db	Darwin, Australia
df	Edwards, USA
et	East Trout Lake, Canada
eu	Eureka, Canada
gm	Garmisch, Germany
hf	Hefei, China
js	Saga, Japan
oc	Lamont, Oklahoma, USA
ll	Lauder, New Zealand
ma	Manaus, Brazil
ny	Ny‐Alesund, Svalbard, Norway
or	Orleans, France
pa	Park Falls, Wisconsin, USA
pr	Paris, France
ra	Reunion Island, France
rj	Rikubetsu, Hokkaido, Japan
so	Sodankyla, Finland
tk	Tsukuba, Ibaraki, Japan
