# Supplementary material for: Refining the Global Picture: The Impact of Increased Resolution on CO2 Atmospheric Inversions Using OCO‐2 XCO2 Retrievals
Source: J Geophys Res Atmos. 2024 Nov 23;129(22):e2024JD041016. doi: 10.1029/2024JD041016 (PMC11585354; doi:10.1029/2024JD041016)
Supplement: Supplementary file 1 — Figure S1 [file JGRD-129-0-s001.pdf]

Journal of Geophysical Research Atmospheres

Supporting Information for

Refining the Global Picture: the Impact of Increased Resolution on CO_2 Atmospheric Inversions using OCO-2 XCO_2 retrievals

Zoé Lloret[1], Frédéric Chevallier[1], Anne Cozic[1]

[1]Laboratoire des Sciences du Climat et de l'Environnement, LSCE/IPSL, CEA-CNRS-UVSQ, Université Paris-Saclay, Gif-sur-Yvette, France

Contents of this file

Figure S1

Introduction

Figure S1 presents an example of a seasonal fit and its different components, taken at TCCON station Reunion. It corresponds to the seasonal decomposition described in detail by the equations in Section 2.3.2 of the manuscript.

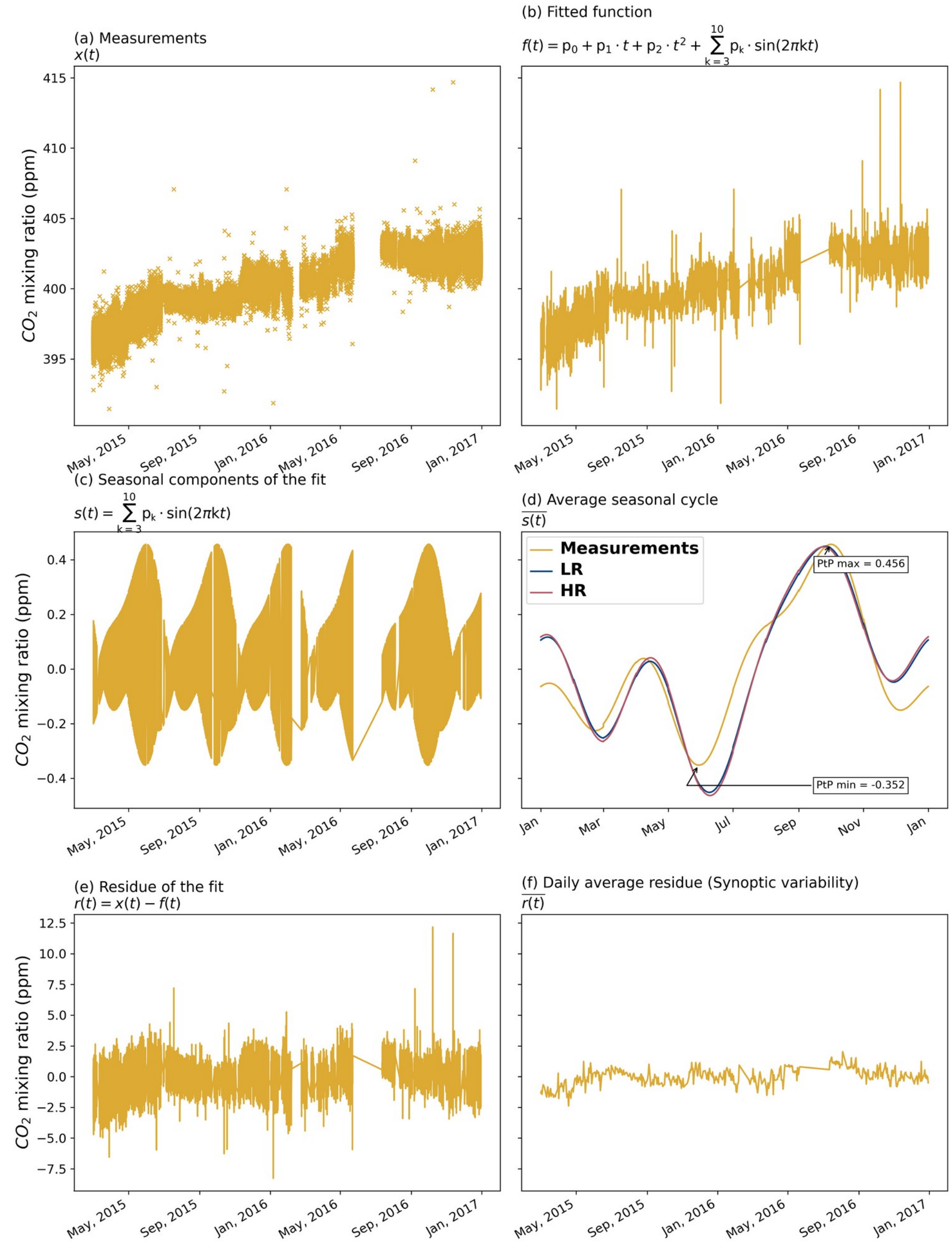

Figure S1. Example of a seasonal fit and its different components, taken at TCCON station Reunion. Only the measurements are fitted, except in panel (d) where the fit of the model at two different resolutions is also plotted. The minimum and maximum values of the peak of the average seasonal cycle of measurements used to calculate the peak-to-peak amplitude are indicated.
